# Composition of soil microbiome along elevation gradients in southwestern highlands of Saudi Arabia

**DOI:** 10.1186/s12866-015-0398-4

**Published:** 2015-03-14

**Authors:** Muhammad Yasir, Esam I Azhar, Imran Khan, Fehmida Bibi, Rnda Baabdullah, Ibrahim A Al-Zahrani, Ahmed K Al-Ghamdi

**Affiliations:** Special Infectious Agents Unit, King Fahd Medical Research Center, King Abdulaziz University, Jeddah, Saudi Arabia; Medical Laboratory Technology Department, Faculty of Applied Medical Sciences, King Abdulaziz University, Jeddah, Saudi Arabia; Biochemistry Department, Faculty of Science, King Abdulaziz University, Jeddah, Saudi Arabia

**Keywords:** Soil bacteria, 16S rRNA, Pyrosequencing, Highlands, Saudi Arabia

## Abstract

**Background:**

Saudi Arabia is mostly barren except the southwestern highlands that are susceptible to environmental changes, a hotspot for biodiversity, but poorly studied for microbial diversity and composition. In this study, 454-pyrosequencing of 16S rRNA gene hypervariable region V6 was used to analyze soil bacterial community along elevation gradients of the southwestern highlands.

**Results:**

In general, lower percentage of total soil organic matter (SOM) and nitrogen were detected in the analyzed soil samples. Total 33 different phyla were identified across the samples, including dominant phyla *Proteobacteria*, *Actinobacteria* and *Acidobacteria*. Representative OTUs were grouped into 329 and 508 different taxa at family and genus level taxonomic classification, respectively. The identified OTUs unique to each sample were very low irrespective of the altitude. Jackknifed principal coordinates analysis (PCoA) revealed, overall differences in the bacterial community were more related to the quantity of specific OTUs than to their diversity among the studied samples.

**Conclusions:**

Bacterial diversity and soil physicochemical properties did not show consistent changes along the elevation gradients. The large number of OTUs shared between the studied samples suggest the presence of a core soil bacterial community in the southwestern highlands of Saudi Arabia.

## Background

The microbial community of soil is extremely diverse and integral part of ecosystems that play a major role in the climate change trough contribution in soil organic matter decomposition [1]. Prime reservoir of global terrestrial carbon resides in soil [2], and fractional scale changes in cycling of total soil carbon could have considerable impacts on the density of atmospheric carbon dioxide. Therefore, a change in soil carbon is a critical regulator of future climate in response to environmental changes [3]. Climate change is a complex phenomenon regulated by numerous factors, including complex interactions and feedbacks between climate, plants, animals and soil microbes [2,3].

Overall, global warming effects are more prime on microbial community and consequent decomposition processes in alpine, arctic and highland regions [3]. However, altitudinal patterns of microbial community have not been well studied and remain poorly understood compared to macroorganisms diversity that have been studied for centuries [4]. Bacterial diversity and abundance probably is reduced with altitude and influenced by ecological and geological factors such as vegetation, temperature and pH level etc. that establish complex interaction [4,5]. Several studies did not observe similar types of consistent changes in microbial community along elevation gradients [6,7]. Species of *Azotobacter chroococcum* and *Azospirillum brasilense* were reported as dominant nitrogen fixing bacteria at high altitude [8]. Seasonal variations have influenced on bacterial community diversity and abundance within taxa in Colorado Mountains. The *Acidobacterium* division was most abundant in spring. Winter community had the highest proportion of *Actinobacteria* and members of the *Cytophaga*/*Flexibacter*/*Bacteroides* division. However, some of the species resist temperature fluctuations, e.g. the α***–****Proteobacteria* [9]. Further studies that parallelly investigate empirical patterns of plants, animals and microorganisms will provide a better sketch of diversity patterns in major environmental gradients of Earth, and will predict system wide ecological responses to climatic changes [4].

Saudi Arabia represents almost 80% of the Arabian Peninsula [10], and at least one**–**third of its land is desert [11]. Saudi Arabia is generally known for high temperature and low rainfall with exception of the southwestern highlands that are covered by diverse forests [12]. According to the report of Darfaoui and Assiri [13], Saudi Arabia is more vulnerable to climate change, and forecasted temperature increase in this region is higher compared to an average increase in the global temperature along with high moisture in the western highlands of the country. So far no study has been conducted to document the soil microbial community in this geographically distinct region with unique environmental conditions. In this study, soil samples were collected from the southwestern highlands of Saudi Arabia at different elevational gradients to document bacterial community composition using high throughput next generation sequencing targeting hypervariable V6 region of 16S rRNA gene. In future prospective, the study would help us to understand the impact of climate change in this region on microbial community composition and their possible consequences on flora and fauna.

## Results

### Physicochemical properties

The studied physicochemical properties were significantly variable within the soil samples except for pH, which ranged from 7.7–8.3 (Table 1). In general, lower percentage of soil organic matter (SOM) and nitrogen were detected in all the analyzed samples range from 0.21–1.75 and 0.02–0.09, respectively. One**-**way ANOVA analysis demonstrated that the concentration of phosphorus was significantly different within the analyzed samples and ranged 5.5–52.1 mg kg^**-**1^.

**Table 1:** **Location of the study sites and physicochemical characteristics of soil samples**

**Sample**	**Location name**	**Elevation (meter)**	**pH**	**Organic matter %**	**Nitrogen %**	**Phosphorus mg/kg**
Swh1	Tabalah	1447	8.3 ± 0.2	0.85 ± 0.03	0.04 ± 0.004	26.4 ± 0.3
Swh2	Tabalah	1447	8.1 ± 0.15	0.71 ± 0.02	0.08 ± 0.001	5.5 ± 0.5
Swh3	Sabt Alalayah	2100	7.7 ± 0.11	1.75 ± 0.01	0.09 ± 0.003	14.4 ± 0.1
Swh4	Sabt Alalayah	2100	8 ± .026	1.75 ± 0.03	0.09 ± 0.001	19.7 ± 0.1
Swh5	Sabt Alalayah	2100	7.8 ± 0.35	1.61 ± 0.01	0.08 ± 0.005	52.1 ± 1.9
Swh6	Sabt Alalayah	2100	7.8 ± 0.05	1.75 ± 0.02	0.09 ± 0.002	48.2 ± 2.0
Swh7	Al Salamah	2312	7.9 ± 0.25	0.7 ± 0.1	0.04 ± 0.001	45.5 ± 0.4
Swh8	Al Salamah	2312	7.8 ± 0.17	1.54 ± 0.04	0.07 ± 0.006	28.2 ± 0.3
Swh9	Afraa	1871	7.8 ± 0.1	0.21 ± 0.06	0.02 ± 0.008	28.2 ± 0.1
Swh10	Afraa	1871	7.9 ± 0.15	1.75 ± 0.04	0.09 ± 0.001	34.9 ± 0.4

### Bacterial community composition

In this study, we obtained a total of 62081 raw sequence reads from 10 samples utilizing Roche 454**-**FLX titanium instruments. After sequence processing, 58617 number of high quality sequence reads (>200 bp) were obtained and assigned to bacteria domain. The average reads number were 5861 ± 1708 sequences per sample.

**Figure 1:**
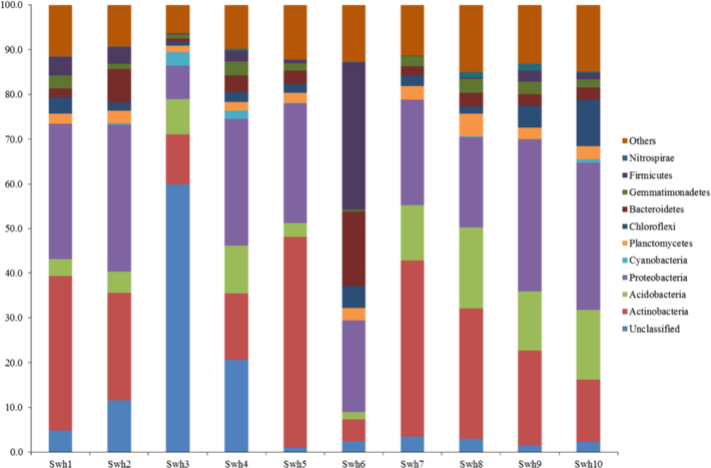
**Inter-samples variability and average relative abundance of the dominant bacterial phyla across soil microbiome collected from different elevational gradients in southwestern highlands of Saudi Arabia.** Others; indicate the collective percentage of minor phyla. Sample numbers showed on the x axis and percentage sequences reads classified on the y-axis.

Bacterial community compositions of the collected soil samples were studied at descending levels of taxonomic classification to find out community membership at different altitudes in the southwestern highlands of Saudi Arabia. Overall, 33 phyla were found across the samples, including a group of unclassified sequences. Twelve phyla were commonly detected within the studied samples. The relative abundances of dominant phyla are presented in Figure 1. Only eight phyla were comprised mean abundance more than 1% in each sample, but they jointly hold 90.4% of the total sequence reads. Most dominant phylum was *Proteobacteria* with mean relative abundance value of 25.7% ± 8.2 and dominantly detected in ≥20% concentration within all samples except from Swh3 (7.6%). Other dominant phyla were *Actinobacteria* and *Acidobacteria* representing bacterial sequences in the range of 4.9–47.1% and 1.5–18.3%, respectively. An average 20.9% of the sequences were unclassified. Concentration of the unclassified sequences were fluctuated in the studied samples, and the sample Swh3 contained highest 65.4% unclassified sequences. There were also identified certain members of *Chloroflexi* (1.7%–10.3%), *Bacteroidetes* (0.8%–16.7%), *Firmicutes* (0.3%–32.9%), *Planctomycetes* (1.5%–5.1%) and *Gemmatimonadetes* (0.8%–3.2%), and were designated as minor phyla detected in lower concentration. Of these groups, *Firmicutes* and *Bacteroidetes* were detected in higher concentration 32.9% and 16.7%, respectively in the sample Swh6 compared to the other samples. The remaining identified phyla/candidate divisions represent much smaller fraction (2.2%) of the bacterial community.

Taxonomic analysis revealed 103 OTUs including a group of 19 (18.4%) unclassified OTUs at class level. Members from *Actinobacteria* (class) dominated the *Actinobacteria* phylum, occupying 17.4% of the total sequence reads, but were showing high variability ranging from 3.9–40.2 % within the samples. Class α***–****Proteobacteria* occupied 12.5% of the total sequence reads and was dominating phylum *Proteobacteria* ranging from 4.3–21.9% within the samples compared to other *Proteobacteria* classes, β*–Proteobacteria* (4.5 ± 2.4%), γ*–Proteobacteria* (3.7 ± 2.1%) and δ*–Proteobacteria* (3.3 ± 1.2 %). Members of the class *Acidobacteria***–**6 (4.0 ± 2.9 %) and *Thermoleophilia* (*Actinobacteria*, 3.5 ± 2.8%) were commonly detected within the samples. Statistically, only the distribution of rarely detected classes, *Solibacteres* (*Acidobabcteria*), *Actinobacteria* MB*2A**–**108 and unclassified class of *Verrucomicrobia* present in <1% concentration of the total sequence reads were significantly different (*p* < 0.05) between the groups of samples collected at elevation of 2000 meter below and above.

**Figure 2:**
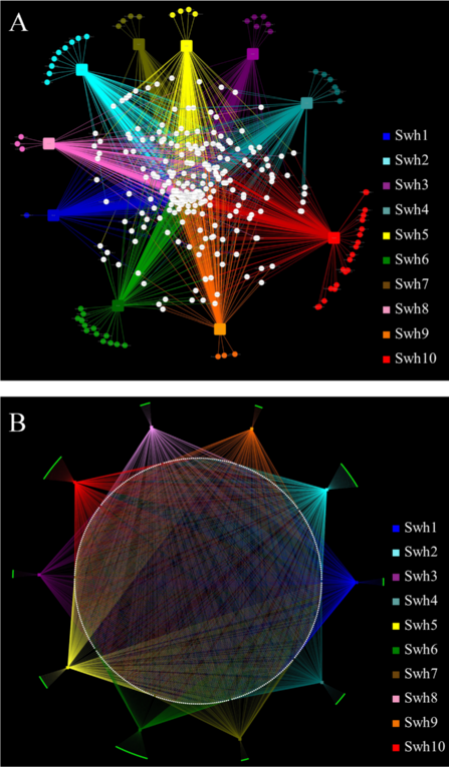
**Networks based analysis of samples/OTUs interaction. (A)** Network showed the connection of OTUs at family and **(B)** genera level among the studied samples. Square nodes represent samples and circle nodes represent the bacterial OTUs. The white circle nodes represent bacterial OTUs commonly found in different samples and connected with more than one edge. Unique bacterial OTUs to a specific samples were connected with a respective sample node by single line.

All the sequence reads were assigned into 329 OTUs at the family level of the taxonomy. Out of 329, the 193 (58.7%) OTUs were unclassified to any define family. Average, 162.1 ± 25.3 OTUs at family level were observed per sample. Fifty seven OTUs were commonly detected within samples and were representing 74% of the total sequence reads. Only few OTUs were present exclusively in each sample with mean relative abundance of 7.0 ± 5.7 ranged (1**–**20). Maximum number of unique family level OTUs were identified in the sample Swh10 and minimum number in Swh1 (Figure 2A). The highest diversity was observed in the sample Swh10 containing 219 taxa at family level followed by Swh4 (183), Swh5 (173) and Swh2 (170). Apart from unclassified taxa OTUs, other dominant taxa at family level were *Actinomycetales* fam. (4.3 ± 3.2), *Micromonosporaceae* (3.1 ± 2.7%), *Geodermatophilaceae* (3.0 ± 3.1%), *Chloracidobacteria* fam. (2.9 ± 2.3%) and *Acidobacteria***–**6 fam. 1 (2.7 ± 2.0). All the dominant families >1% mean concentration were commonly present within the studied samples except from *Clostridiaceae* (1.2 ± 3.3%) that was detected at 10.5% in sample Swh6 and present at a rare concentration in the other 4 samples Swh4, Swh5, Swh9 and Swh10.

Overall, 508 OTUs were found at genus level taxonomic classification in the total sequence reads. Among those, only 164 OTUs were assigned to defined genera. The 54 OTUs were commonly detected within the analyzed samples and were representing 66.3% of the total sequence reads. Only few OTUs were present specifically per studied samples ranged from 6–26. Maximum number of unique 26 OTUs at genus level were identified in the sample Swh6 (Figure 2B). On average, 227.6 ± 41.1 OTUs at genus level were identified per sample, and highest diversity was observed in the sample Swh10 containing 296 taxa followed by Swh5 (268) and Swh2 (265). The genera from following groups *Actinomycetales*, *Chloracidobacteria*, *Geodermatophilaceae*, *Acidobacteria***–**6, *Rhizobiales*, *Betaproteobacteria*, *Micromonosporaceae*, *Solirubrobacterales*, *Alphaproteobacteria* and *Pseudonocardia* were dominantly detected at level of >1% concentration in each sample.

### Pyrosequencing data statistical analyses

**Figure 3:**
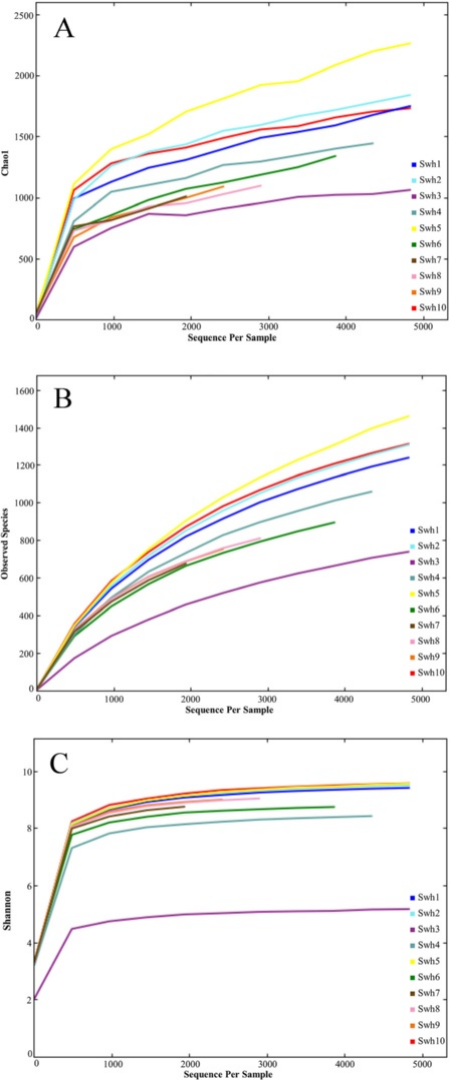
**Alpha diversity of the sequence reads from soil samples collected along elevational gradients from southwestern highland of Saudi Arabia. (A)** Chao1 index, **(B)** rarefaction analysis of the observed species and **(C)** The Shannon index were computed.

Chao1 index, rarefaction and the Shannon index were calculated to estimate the alpha diversity. Total 4603 OTUs (defined using a 97% cut off value) were found in the complete data set of processed sequence reads. Chao1 analysis revealed a decreased trend of richness in the samples Swh3, Swh8, Swh9 and Swh7 compared to the samples Swh5, Swh2, Swh1 and Swh10 (Figure 3A). The highest richness value of Chao1 was found in sample Swh5 (2262) and lowest in Swh3 (1060). In rarefaction curves, all the studied samples were tended to approach saturation plateau, and the samples Swh5, Shw10, Swh2 and Swh1 were plotted in the upper part of the graph (Figure 3B). The rarefaction curve indicated variation in OTUs density within the soil samples collected from different altitudes and locations, but the sequence coverage was still sufficient to capture the diversity of bacterial community. The Shannon Wiener index calculated at 3% dissimilarity showed the lowest value of evenness (4.5) for the sample Swh3 compared to the all other samples that revealed the highest value of evenness (>8.0, Figure 3C). Statistically, no significant difference was observed in the Choa1 and the Shannon Wiener index between the groups of samples collected at different altitudes.

**Figure 4:**
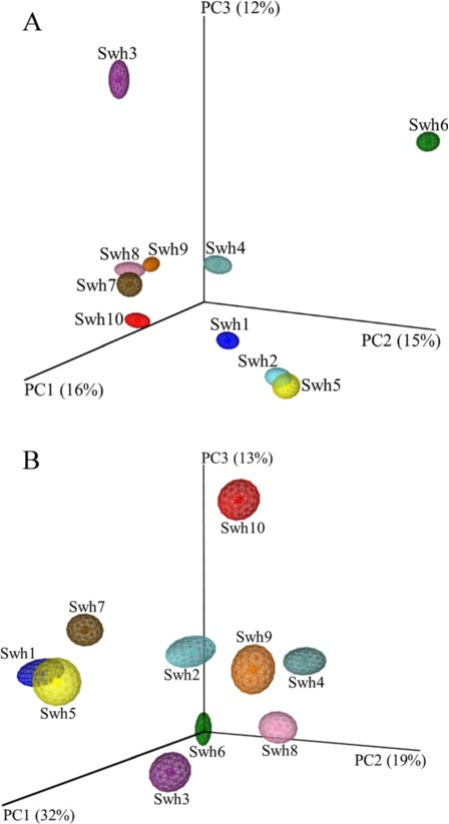
**Principal coordinates analysis (PCoA) of bacterial communities in different soil samples collected along elevational gradients from southwestern highlands of Saudi Arabia. (A)** Unweighted PCoA explained 43%variation and **(B)** weighted PCoA explained 64% of variation along the first three axes. Ellipses around center points represent the interquartile range.

Jackknifed PCoA was performed using Unifrac metrics to compare overall composition of bacterial community within the samples and to cluster the bacterial community along axes of maximal variance according to their diversity (qualitative/unweighted) and concentration (quantitative/weighted). Jackknifed unweighted PCoA explained relatively small variation (43%) along the first three axes (Figure 4A). Jackknifed weighted PCoA explained 64% of variation along the first three axes demonstrating that overall differences between bacterial communities within the samples were more related to the richness of specific taxa OTUs compared to their presence or absence (Figure 4B). In the three-dimensional plot visualized from Unifrac unweighted distance matrix PCoA, samples Swh7, Swh8, Swh9 grouped in one cluster and samples Swh1, Swh2 and Swh5 made another cluster. The other four samples were scattered in the plot. The samples were broadly distributed along PC1, PC2 and PC3 in the weighted distance matrix principle coordinate plot except for the sample Shw1 and Swh5.

## Discussion

Natural ecosystem in the western highlands of Saudi Arabia is susceptible to climate change as result of global warming, overgrazing and expansion of uncontrolled urbanization that is leading to changes in species composition, richness and a decrease in biodiversity [10,13]. Presently, western highlands of Saudi Arabia are experiencing a drastic shift from ruler to urban environment and population influx. Urbanization has detrimental effects on soil ecosystems and microbial community [14]. According to GCM models, expected average warming in Saudi Arabia for the year 2041 will be higher than the global average [13]. Therefore, it is critical to document the ecosystem, particularly the poorly studied microbial community over different parts of the country and to plan for the sustaining of local ecosystems in the western highlands.

We investigated the soil bacterial community composition and soil residues along an altitude in the southwestern highlands of Saudi Arabia, where the ecosystem is expected to be more fragile with respect to climate change [3,15]. We found that neither bacterial diversity nor soil physicochemical properties showed consistent changes with altitude. There were no significant differences observed in pH level at different altitudes. Similarly, Tukey HSD analysis indicated that the concentration of SOM and total nitrogen were not significantly different within the samples Swh3, Swh4 and Swh10 collected at different altitudes. Previously, it was reported that microbial flora does not affect by altitude. Physicochemical factors such as SOM, total nitrogen, electrical conductivity and pH changed with altitude [16]. The relation between SOM and altitude increased linearly in grassland soil [17]. Generally, effect of altitude on soil chemistry is dependent on vegetation, climate and environmental ecology of the investigated study site [3,7]. Overall, the concentration of SOM was less than 2% within each analyzed soil samples that limited to desert area and representing low**–**lying of SOM contents in the southwestern highlands of Saudi Arabia.

In order to understand bacterial diversity and community’s composition at southwestern highlands, we analyzed the output of 16S rRNA gene sequence reads qualitatively and quantitatively. The identified OTUs unique to each sample were very low irrespective of the altitudinal level. The highest number of twenty six unique OTUs at genus level were identified in the sample Swh6 collected at 2100 meter height followed by 22 unique OTUs identified in the Swh10 collected at 1871 meter height. Maximum diversity was detected in the samples Swh10, Swh5 and Swh2, respectively collected at different altitudinal level. Consistent with several previous studies, soil from the southwestern highlands at different altitudes were dominated by the conserved set of phyla *Proteobacteria*, *Actinobacteria* and *Acidobacteria* that were commonly detected in different types of soil such as agriculture, forest and Arctic [18-20]. It suggests the presence of a well adapted soil core bacterial community that is resistant to environmental disturbance factors and commonly present in different types of soil. However, the overall similarities and dissimilarities are associated with the soil type acting as a dominant factor driving bacterial community composition [21]. For example, phyla *Firmicutes* and *Bacteroidetes* were detected at higher concentrations in sample Swh6 compared to the other samples. This difference may be attributed to the sedimentary nature of soil collected around a lake and vegetation of the sampling site. Similar structure in microbial community was detected along different alpine vegetative zones and altitudinal gradients from 900–1900 meters in the Austrian Limestone Alps [6], and in the soil from the Bornean tropical forest at altitudinal gradients (700–2700 meters) [22]. Margesin et al. [23] reported a decrease in microbial activity and shift in microbial community composition with altitude in the Grossglockner mountain area of the Austrian Central Alps. Probably, the apparent inconsistency between the studies may be due to difference in the study sties, climate, elevation increments and vegetation or methodology used for assessment of microbial community composition. Several researchers identified significant influence of vegetation and plant species over microbial flora [24] and soil chemistry [25]. In this study, we did not focus on the association of plant species specific interaction with microbial community at the sampling sites that were mainly covered by the scattered density of *Juniperus procera* followed by *Acacia* spp. *Olea europaea* and shrubs. Ushio et al. [26] revealed the indirect role of plant species in regulation of soil microbial community composition mainly through their effects on soil physicochemical properties such as pH, total carbon and nitrogen etc. The interaction between plants, soil chemistry, microorganism and other ecological factors is quite complex phenomena that drives the ecosystem functions and any alteration in these factors might affect the microbial community composition and the ecological processes [3,22,27].

Interestingly, the majority of sequence reads from all the analyzed samples were not classified to any defined taxonomy units at and below family level. It is consistent with the previous studies investigated untouched environments such as Antarctica, and found a unique type of flora that showed lower similarities with 16S rRNA gene sequences retrieved from other environmental samples [17]. It should be noted, there are few limitations exist in our study that include selection of few sampling sites as a proxy to assess the western highland of Saudi Arabia, although the studied sites have a representative landscape of southwestern highlands. Our results based on single time point sampling and seasonal dynamics might affect the microbial community’s diversity and composition. In fact, some previous studies have noticed that long**–**term patterns of subsurface microbial community are likely to remain generally intact [28].

## Conclusions

Analysis indicated that bacterial community within soil samples collected from southwestern highlands of Saudi Arabia were similar but not identical, and did not show a consistent shift with altitudinal level. Overall, 12 phyla were commonly found across the samples and a group of core dominant phyla *Proteobacteria*, *Actinobacteria* and *Acidobacteria* reported previously from other different types of soil. Members from the phyla *Chloroflexi*, *Bacteroidetes*, *Firmicutes*, *Planctomycetes* and *Gemmatimonadetes* were present in lower abundance. PCoA analysis revealed, overall differences between the samples were more related to the abundance of specific OTUs than to their presence or absence. There is need of further studies to investigate the microbial community in conjunction with other ecological parameters in the western highlands of Saudi Arabia. Because it may encounter catastrophic shifts due to urbanization and climate change.

## Methods

### Study area and samples collection

**Figure 5:**
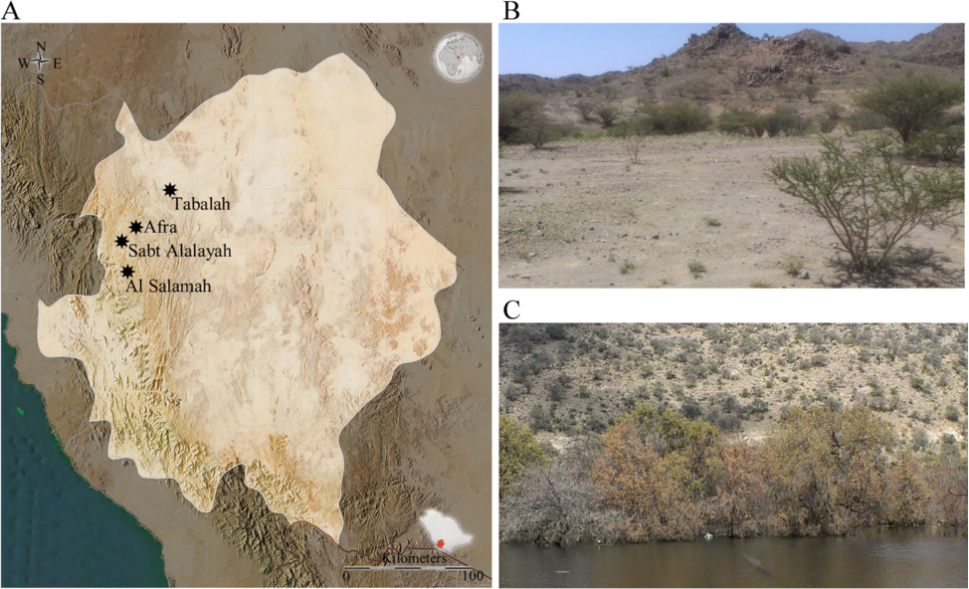
**Map of the Asir region and studied sites. (A)** Locations of soil sampling from selected four studied sites along different elevational gradients in southwestern highlands of Saudi Arabia were marked with asterisk (∗). **(B)** Photographic images of sampling sites from the Tabalah and **(C)** the Sabt Alalayah. Map of the Asir region was reproduced from maphill (http://www.maphill.com).

Saudi Arabia is mostly an arid country with a few exceptional sub**–**humid areas including southwestern highlands in the Asir region that occupies 4.3% of the total country area and located between latitudes 17–21°N and longitudes 41–45°E [29]. Annual rainfall in this region is in the range of 300**–**500 millimeters and summer temperature raises up to 30°C and above that comparatively higher from other highlands of the world [12]. This region is well known for natural vegetation and for being a plants biodiversity hotspot in the Arabian Peninsula [12]. In this study, soil samples were collected at a broad level of elevational gradients having different density of vegetation and flora (Figure 5). Sandy type of soil samples, Shw1 and Swh2, were collected from the Tabalah area (19°52′55.7″N, 42°09′03.8″E) having no human activity or animal influence and low density of vegetation. Samples Swh3**–**Swh6 were collected from the Sabt Alalayah around the Shibanah wild park (19° 34′ 46.87″ N, 41° 55′ 14.19″ E) vegetated mainly by *Olea europaea*, *Tarchonanthus camphorates* and shrubs. Sample Swh6 was collected around a lack of rainwater, and Swh3 was collected from the land covered by mosses. Sample Swh7 and Swh8 were collected in the high mountainous area of Al Salamah (19°24′34.3″N, 42°00′29.7″E) covered by scattered condensed vegetation. Samples Swh9 and Swh10 were collected from the Afraa area (19°41′23.3″N, 42°01′33.4″E) vegetated by shrubs, *Olea europaea* and *Juniperus procera* etc.

At each sampling spot, quadrates of 3.5 × 3.5 meters were selected. Surface soil of 3 cm was removed. Multiple samples were collected from the four corners and middle of each quadrate at depth of 10 cm with a sterilized spatula, and the samples were sieved through 2 mm mesh. Samples from each spot were mixed in a sterilized bag and frozen at **–**80°C for DNA extraction. All the samples were collected on the same day in May 2012.

### Chemical analysis

The pH of each sample was measured using Meridian benchtop meters (Denver, Germany) in a saturated colloid solution of soil in deionized water. Total soil organic matter (SOM) was determined using the partial oxidation method. Total phosphorus was measured colorimetrically and total nitrogen (TN) was analyzed by the micro Kjeldahl method [30]. Each sample was analyzed in triplicate.

### DNA extraction and pyrosequencing

Samples were homogenized and metagenomic DNA isolation was performed using PowerSoil® DNA extraction kit (MoBio Laboratories, Carlsbad), as recommended by the manufacturer. To get maximum coverage, DNA extraction from each sample was performed in triplicates, pooled and quantified using Qubit fluorometer (Invitrogen, USA). PCR amplification of 16 S rRNA gene was performed from extracted DNA of each sample by using bar**–**coded 926 F AAACTYAAAKGAATTGACGG and 1394R ACGGGCGGTGTGTRC universal primers containing the A and B sequencing adaptors targeting hypervariable V6–V8 region of 16S rRNA gene following the procedure of Dowd et al. [31]. Amplicon products of PCR from all samples were quantified using high sensitivity Qubit technology and purified using Agencourt Ampure beads (Agencourt, USA). Sequencing was performed on 454 FLX**–**titanium amplicon pyrosequencing technology (Roche, Basel Switzerland) following the manufacturer’s protocol.

The raw sequence data was processed using a proprietary analysis pipeline (MR DNA, TX USA). The sequence reads containing N, sequences with homopolymer runs exceeding 6 bp, chimera and sequence reads < 200 bp were removed. Sequences were depleted from barcodes and primers. The high quality sequence reads were clustered into operational taxonomic units (OTUs) using threshold of 97% sequence similarity. The OTUs were taxonomically classified using BLASTn against a curated GreenGenes database, and RDP classifier and RDP training set [32].

### Statistical analysis

Richness and biodiversity index of the OTUs were calculated with implementation of Chao1 and non**–**parametric Shannon formula using QIIME v1.8 software package [33]. The UniFrac unweighted pair group method with arithmetic mean phylogeny**–**based distance metric analysis was used to investigate differences in microbial community among soil samples collected at different altitudes from the southwestern highlands of Saudi Arabia [34]. The resulting matrices were also processed for PCoA that showed fraction of total variance at each axis. One**–**way anova and Tukey HSD (Honestly Significant Difference) tests were used to statistically compare physicochemical parameters, and no**–**parametric Kruskal**–**Wallis along with Mann**–**Whitney analysis were performed to identify significantly different bacterial taxa between the samples collected below and above 2000 meters. Statistical analyses were performed using SPSS version 20.

### Availability of supporting data

Sequence data of this study is available in the NCBI Sequence Read Archive under accession no. SRX759755.
